# Diagnostic challenges in biliary cytology of biliary inflammatory pseudotumor of the liver: A case report and a review of the literature

**DOI:** 10.3892/mi.2026.303

**Published:** 2026-02-18

**Authors:** Yumiko Higuchi, Toshihiko Kato, Eishin Kurihara, Tomonari Toyama, Takuya Fusegawa, Mami Ubukata, Tomiko Yoshizawa, Tomohiro Imaizumi, Akiko Suzuki, Mei Oshima, Fuki Endo, Shun Matsumoto, Katsuhiro Hagiwara, Yosuke Otake, Shiro Sugihara, Noriyuki Tani, Yasuo Imai

**Affiliations:** 1 Department of Diagnostic Pathology, Ota Memorial Hospital, SUBARU Health Insurance Society, Ota, Gunma 373-8585, Japan; 2 Department of Gastroenterology, Ota Memorial Hospital, SUBARU Health Insurance Society, Ota, Gunma 373-8585, Japan; 3 Department of Clinical Laboratory, Gunma Prefectural Cancer Center, Ota, Gunma 373-8550, Japan; 4 Department of Central Clinical Laboratory, Kiryu Kosei General Hospital, Kiryu, Gunma 376-0024, Japan; 5 Department of Surgery, Ota Memorial Hospital, SUBARU Health Insurance Society, Ota, Gunma 373-8585, Japan

**Keywords:** biliary inflammatory pseudotumor, liver, cholangiocarcinoma, ENBD bile cytology, tumor marker, percutaneous core needle biopsy

## Abstract

Biliary inflammatory pseudotumor (IPT) of the liver is a rare benign tumor of which radiographic images, such as biliary stricture and dilatation, resemble those of cholangiocarcinoma (CC). Although repeated biliary cytology via endoscopic nasobiliary drainage (ENBD) is performed for differential diagnosis, a correct diagnosis remains difficult. The present study describes the case of a 76-year-old female patient with chronic liver disease. During surveillance, intrahepatic CC was suspected by imaging analyses; however, the carcinoembryonic antigen and CA19-9 levels were within normal ranges. Percutaneous core needle biopsy was not performed due to technical reasons; however, a left lobectomy was performed based on the diagnosis of malignancy by ENBD bile cytology. A pathological examination of the resected liver demonstrated biliary IPT. Reflecting on the cytological diagnosis of this case, a multi-reviewer re-evaluation of the biliary cytology of this IPT case and 2 cases of well-differentiated CC was then performed, which demonstrated difficulty in distinguishing between them. On the whole, ENBD cytology can play a limited role in differentiation between benign and malignant bile strictures. Surgical indication should be carefully determined and percutaneous core needle biopsy may be warranted in patients with normal tumor marker levels.

## Introduction

Inflammatory pseudotumor (IPT) represents a heterogenous group of mass-forming lesions that involve various organs and is characterized by prominent inflammatory infiltrates (1). Several lesions previously considered to be IPT have been presently identified as different entities. The neoplastic variants of IPT include inflammatory myofibroblastic tumor that is associated with anaplastic lymphoma kinase (ALK) translocation and inflammatory pseudotumor-like follicular dendritic cell tumors of the liver and spleen that are related to clonal Epstein-Barr virus infection (1). These lesions turned out to be true neoplasms. In addition, there exist infectious and autoimmune-induced IPTs, such as mycobacterial spindle-cell IPT of lymph nodes and immunoglobulin (Ig) G4-related tumefactive lesions, respectively (1). The etiology and pathogenesis remain unclear for a subset of IPTs that lack the entities described above.

In the liver, IPT is occasionally encountered when a biopsy or resection of mass lesions is performed due to clinical concerns of primary or metastatic liver tumors. In a previous study of resected focal lesions in 403 patients, the incidence of hepatic IPT was reported to be 0.7% (2). The majority of IPTs are presumed to be an exuberant response to cholangitis or infection, although in most cases, the infectious agent is unknown (3). Hepatic IPTs are classified into two types in terms of tumor location, that is, parenchymal IPT and biliary IPT (4). The diagnosis of hepatic parenchymal IPT is often possible following imaging-directed percutaneous core needle biopsy, while this is not the case with hepatic biliary IPT. Obstructive jaundice, biliary stricture in endoscopic retrograde cholangiopancreatography (ERCP), and cellular atypia that is frequently documented at preoperative biliary cytology may suggest cholangiocarcinoma (CC), leading to unnecessary surgery.

The present study descries a rare case of hepatic biliary IPT in a patient who had been misdiagnosed with CC pre-operatively based on the repeated biliary cytology through endoscopic nasobiliary drainage (ENBD) and then underwent left lobectomy. In addition, issues encountered with the cytological diagnosis of this case are discussed by comparing the present case with 2 cases of well-differentiated CC. To the best of our knowledge, this is the first report to validate the limitations of biliary cytology of hepatic biliary IPT by a multi-reviewer re-evaluation.

## Case report

**Figure 1:**
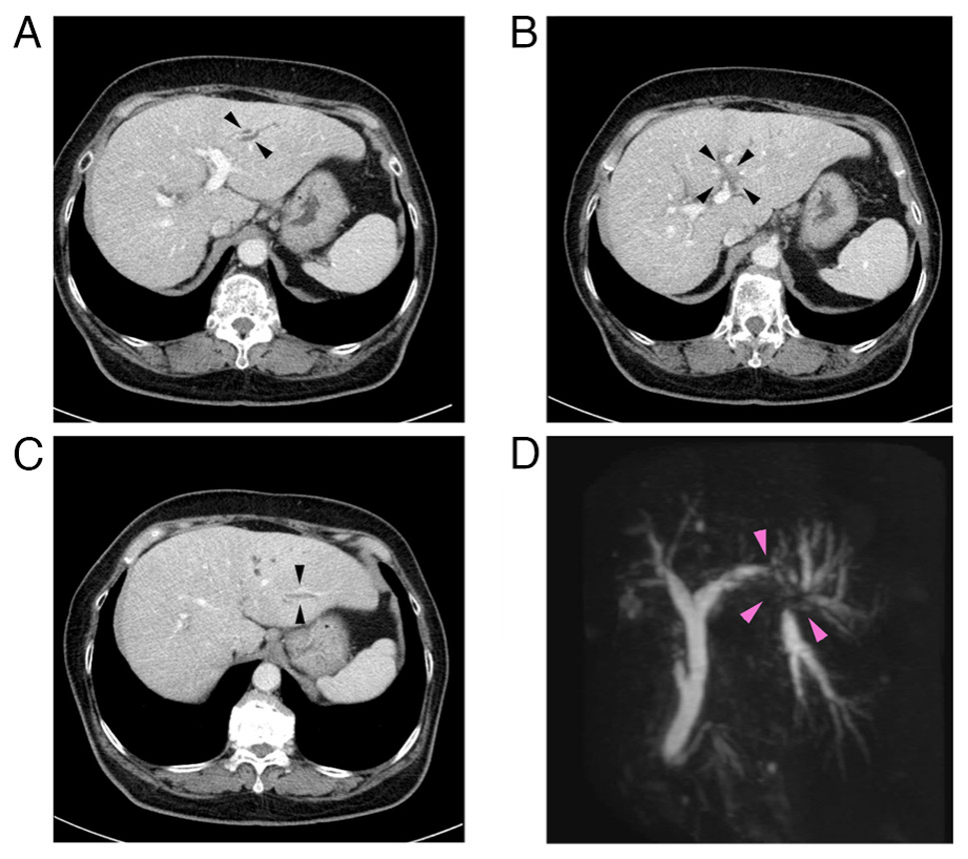
Imaging studies. (A) CECT in the portal phase demonstrated the dilatation of the B3 bile duct. (B) CECT in the portal phase demonstrated a 1x1 cm-sized low density lesion adjacent to the umbilical portion of the left portal vein. (C) CECT in the portal phase demonstrated dilatation of the B2 bile duct. (D) Magnetic resonance cholangiopancreatography revealed stricture and separation of the left branch of intrahepatic bile duct at the bifurcation of B2 and B3 bile ducts (arrows). CECT, contrast-enhanced computed tomography.

In 2025, a 76-year-old woman, who had been under observation for diabetes and liver dysfunction, was referred to the Department of Gastroenterology, Ota Memorial Hospital, Ota, Japan, due to dilatation of the bile duct and portal vein (P3) obstruction in the left lobe of the liver by contrast-enhanced computed tomography (CECT). Laboratory tests at the first visit were performed at the Department of Clinical Laboratory, Ota Memorial Hospital, as routine testing, and the results were the following: White blood cell count, 7,230/µl (reference range, 3,500-8,500/µl); red blood cell count, 514x10^4^/µl (reference range, 380-520x10^4^/µl); hemoglobin, 15.0 g/dl (reference range, 11.5-15.5 g/dl); platelets, 23.6x10^4^/µl (reference range, 12.0-33.0x10^4^/µl); total bilirubin, 0.74 mg/dl (reference range, 0.4-1.5 mg/dl); aspartate aminotransferase, 32 U/l (reference range, 13-30 U/l); alanine aminotransferase, 27 U/l (reference range, 7-23 U/l); alkaline phosphatase, 200 U/l (reference range, 38-113 U/l); γ-glutamyl transferase, 215 U/l (reference range, 9-32 U/l); HbA1c (NGSP), 6.3% (reference range, 4.6-6.2%); IgG, 1,228 mg/dl (reference range, 861-1,747 mg/dl); carcinoembryonic antigen (CEA), 2.8 ng/ml (reference range, 0.0-5.0 ng/ml); CA19-9, <2.0 U/ml (reference range, 0.0-37.0 U/ml); alpha-fetoprotein, 2.3 ng/ml (reference range, 0.0-10.0 ng/ml); PIVKA-2, 20.47 mAU/ml (reference range, 0.0-39.0 mAU/ml); HBs-Ag (-), HCV-Ab (-), anti-nuclear antibody (-) and anti-mitochondrial antibody (-). A CECT scan revealed an ~1 cm-sized low-density lesion adjacent to the umbilical portion of the left portal vein with peripheral bile duct dilatation in the lateral segment of the liver (Fig. 1A-C). Magnetic resonance cholangiopancreatography revealed the disruption of the bile duct at the bifurcation of B2 and B3 bile ducts (arrows) (Fig. 1D). The ERCP findings demonstrated severe stenosis at the origin of the B3 bile duct, which prevented fine needle aspiration. ENBD tube was placed in the B2 bile duct and serial bile cytology was performed. Samples were prepared with BD SurePath^TM^ liquid-based cytology (SP-LBC) (Becton, Dickinson and Company). In brief, bile juice was centrifuged at 1,400 x g for 5 min at room temperature. The cell pellet was fixed with Cytorich^TM^ Red (Becton Dickinson) overnight and then washed. The cells were deposited onto BD SurePath^TM^ Precoated slides (Becton, Dickinson and Company) according to the manufacturer's instructions. Following emersion in 95% ethanol, the slides were subject to Papanicoloau staining with Tissue-Tek Prima^®^ Plus (Sakura Finetek) according to the manufacturer's instructions. The stained glass slides were inspected under a light microscope (BX53; Olympus Corporation).

**Figure 2:**
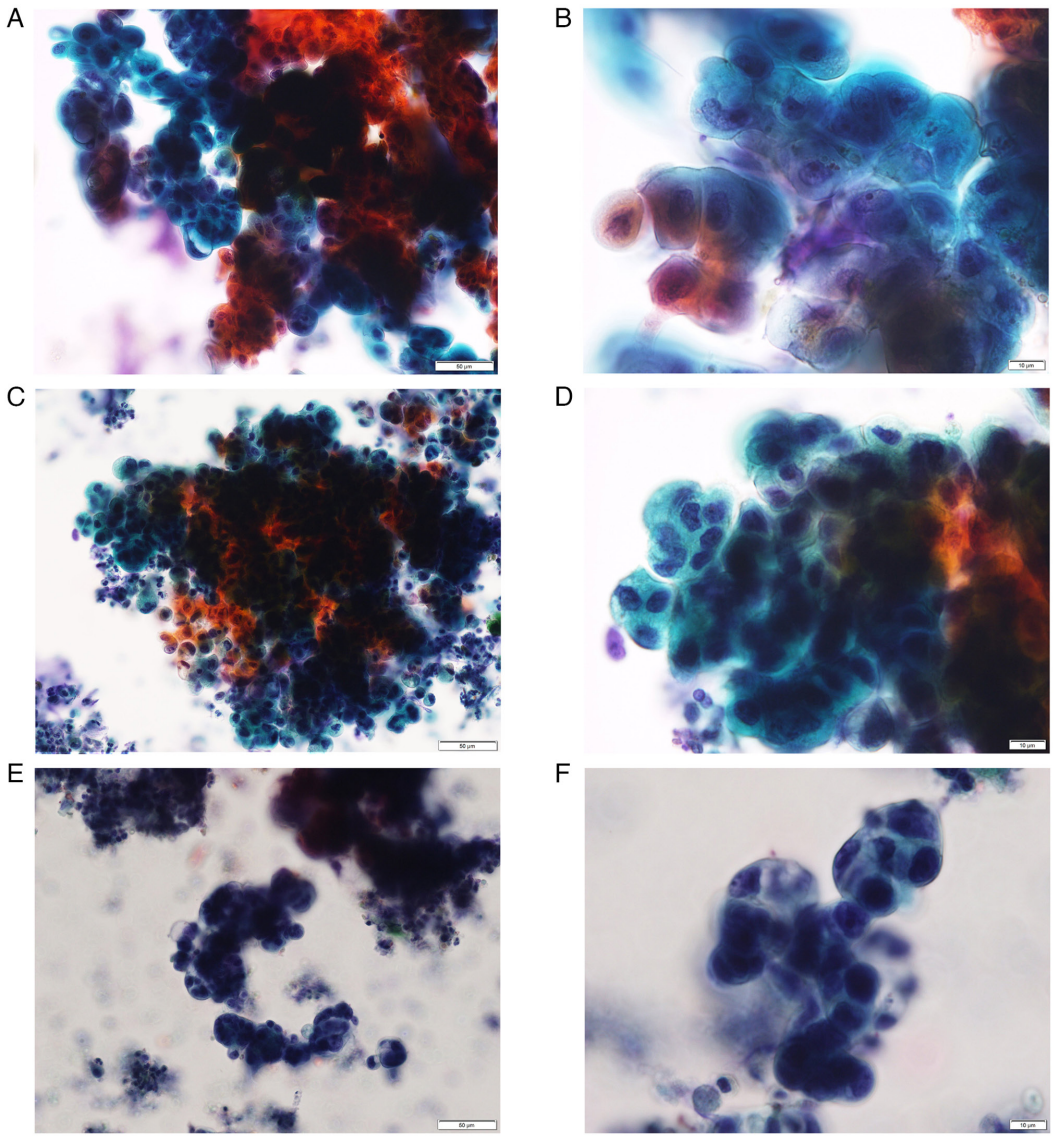
Cytomorphology of the present IPT case and 2 cases of well-differentiated CC for comparison. (A) Bile cytology from the present IPT case (case 1). Large clusters of cells presenting irregularly overlapped nuclei, irregularly arranged nuclei and irregular cluster margins, suggesting malignancy based on the diagnostic criteria for bile cytology by the Japanese Society of Clinical Cytology 2015 (Pap; magnification, x20). (B) Bile cytology from the present IPT case (case 1). Enlarged nuclei, irregular shaped nuclei and nucleoli swelling in the small clusters were also compatible with malignancy (Pap; magnification, x60). (C) Bile cytology from a 67-year-old male patient with CC (case 2). Large clusters, demonstrating similar findings with those of Case 1 (Pap; magnification, x20). (D) Bile cytology from case 2. Small clusters, demontstrating similar findings with those of case 1. Hyperchromasia may be more prominent than case 1 (Pap; magnification, x60). (E) Bile cytology from a 70-year-old male patient with CC (case 3). Large clusters, demonstrating similar findings with those of case 1 (Pap; magnification, x20). (F) Bile cytology from case 3. Small clusters, demonstrating similar findings with those of case 1. Hyperchromasia may be more prominent than case 1 (Pap; magnification, x60). IPT, inflammatory pseudotumor; CC, cholangiocarcinoma; Pap, Papanicoloau.

**Figure 3:**
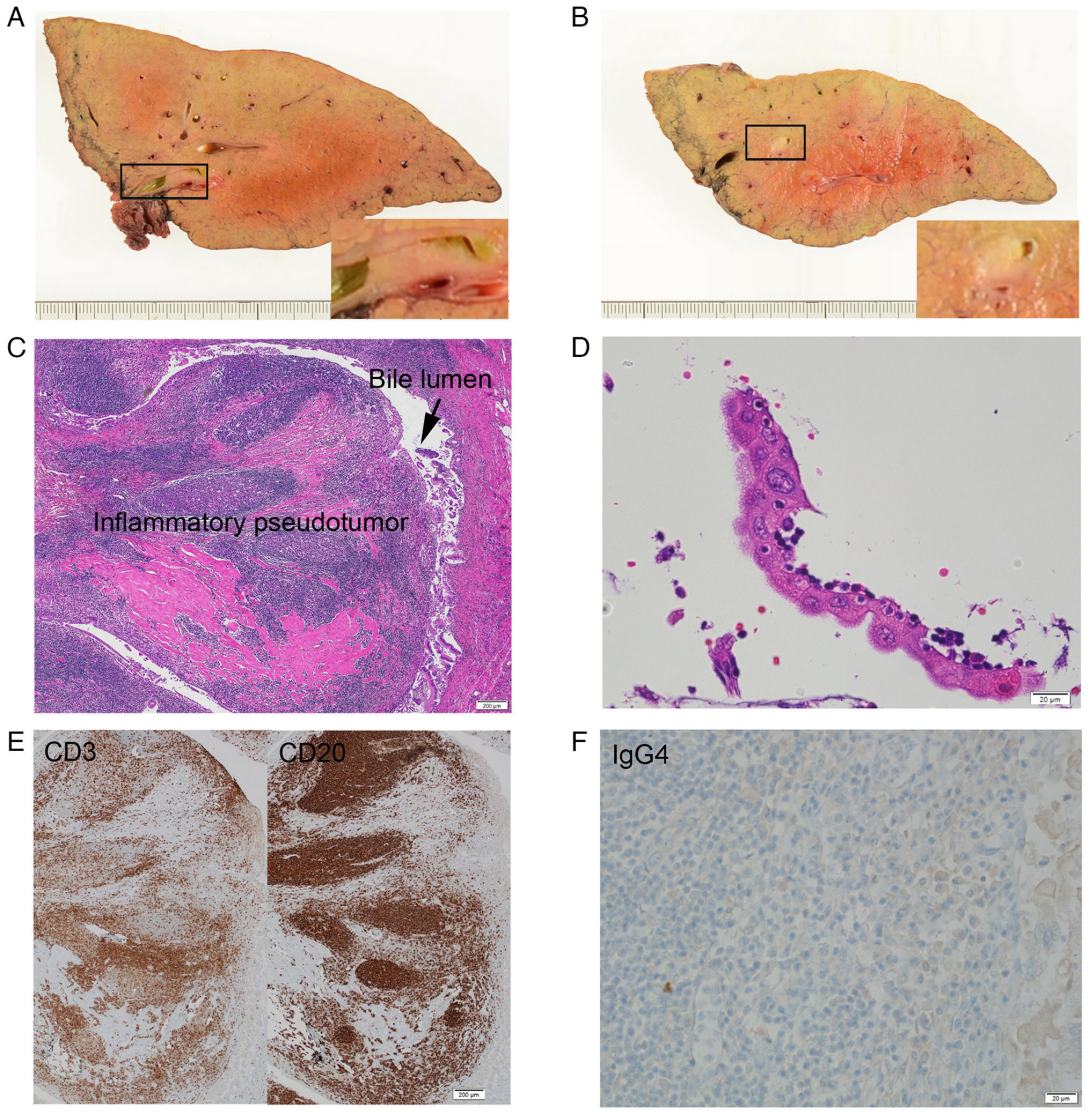
Pathological examination of the resected liver. (A) Cut surface of the resected left lobe of the liver demonstrated yellowish-white tumors adjacent to the B3 bile duct, which caused stenosis of the bile duct. (B) Cut surface of the resected left lobe of the liver demonstrated yellowish-white tumors adjacent to the B2 bile duct, which caused stenosis of the bile duct. (C) The tumor consisted of mature lymphoid tissue with hyalinized collagen deposit, which was diagnosed as biliary IPT (H&E; magnification, x4). (D) The overlying bile eithelium revealed strong reactive atypia, such as enlarged nuclei, anisokaryosis and prominent nucleoli (H&E; magnification, x40). (E) This IPT consisted of both CD3-positive (left panel) and CD20-positive (right panel) cells, suggesting a reactive lesion (magnification, x4). (F) There were few IgG4-positive cells in this IPT (magnification, x40). H&E, hematoxylin and eosin; IPT, inflammatory pseudotumor.

Cytologically abnormal findings, such as irregular nuclear overlapping, irregular cluster margins, and irregular nuclear arrangement were observed against an inflammatory background primarily composed of numerous neutrophils (Fig. 2A and B). These finding met the criteria of Cytology Guidelines 5, Digestive System, edited by the Japanese Society of Clinical Cytology for adenocarcinoma (Table I) (5,6), and a diagnosis of intrahepatic CC was made. A left hepatic lobectomy was performed, and the cut surface of the resected liver revealed multiple yellowish-white polypoid tumors, up to 8x5 mm in size, which obstructed the B2 and B3 bile ducts (Fig. 3A and B).

The resected specimen was routinely processed for pathological diagnosis at the Department of Pathology, Ota Memorial Hospital. In brief, the specimen was fixed with 10% neutral buffered formalin for 48 h at room temperature and then embedded in paraffin. The subsequent 3 µm-thick sections were stained with hematoxylin and eosin (H&E) with Tissue-Tek Prima^®^ Plus (Sakura Finetek) for 55 min at room temperature according to the manufacturer's instructions. The immunohistochemical staining of CD3 (clone 2GV6; cat. no. 518110079; Roche Diagnostics), CD20 (clone L26; cat. no. 518110086; Roche Diagnostics) and IgG4 (clone MRQ-44; cat. no. 06523854001; Roche Diagnostics) was outsourced to SRL Central Laboratory. The stained glass slides were inspected under a light microscope (BX53; Olympus Corporation).

Histologically, the polypoid tumor protruded into the bile lumen (Fig. 3C). With the background of micronodular liver cirrhosis, dense lymphocyte and plasma cell infiltration were observed under the bile duct epithelium, accompanied by the formation of multiple lymphoid follicles and the dense collagen deposit with hyalinization (Fig. 3C). Eosinophilic infiltration was not remarkable. Although the portal vein was compressed by the lymphoid tissue, no obliterative phlebitis was observed. Portions of the biliary epithelium exhibited severe atypia, such as nuclear enlargement, anisokaryosis and swollen nucleoli (Fig. 3D), but no interstitial infiltration was observed. The lymphoid tissue comprised similar amounts of CD3-positive T cells and CD20-positive B cells, suggesting reactive lesion (Fig. 3E). The histological final diagnosis was lymphoplasmacytic pseudotumor. Immunostaining revealed few IgG4-positive cells, and the lesion did not meet the criteria for IgG4-related disease (Fig. 3F) (7).

Reflecting on the cytological diagnosis before surgery, three board-certified cytotechnologists (YH, TK and TT) and two board-certified cytopathologists (SS and YI) at Ota Memorial Hospital and five board-certified cytotechnologists (TF, MU, TY, TI and AS) from neighboring institutions blindly re-evaluated the ENBD cytology specimens prepared with SP-LBC from this case (case 1) (Fig. 2A and B) and two cases of well-differentiated CC at the hepatic hilus (cases 2 and 3) (Fig. 2C, D, E and F), which had been histologically confirmed following surgical resection at Ota Memorial Hospital in 2024. Well-differentiated CC was selected for comparison, as poorly differentiated CC is typically not a diagnostic challenge, but well-differentiated CC is difficult to distinguish from benign and/or reactive changes cytologically. Glass slides of the three cases were re-evaluated simultaneously. Although the rate of diagnosis as malignant was somewhat lower in case 1 than those in cases 2 and 3, five investigators (50%) evaluated case 1 as malignant (Table II). There were no cases in which all observers agreed on the diagnosis.

The authors then presented this IPT case in a slide conference at the 39th Annual Meeting of the Kanto Society of Clinical Cytology held in Shizuoka prefecture, Japan in 2025(8). Virtual slides of this case were posted on the society's website 1 month prior to the conference, and board-certified cytotechnologists affiliated with the society voted on several possible diagnoses. Of the 42 respondents, 61.9% selected adenocarcinoma and 23.8% selected other types of cancer, such as adenosquamous carcinoma and hepatocellular carcinoma, while only 14.3% selected regenerative atypia.

## Discussion

IPT is a term used to describe a benign and rare process found in almost every site of the body. Histologically, an IPT contains cells associated with both acute and chronic inflammation, including lymphocytes, plasma cells, myofibroblastic cells and a fibrous reaction. The cause of IPT is unknown, although it is hypothesized that the causes may be trauma, surgical inflammation, autoimmune condition and low-grade mesenchymal tumor. Some IPTs have been associated with IgG4-related sclerosing disease, such as autoimmune pancreatitis, sclerosing cholangitis, sialoadenitis, retroperitoneal fibrosis, tubulointerstitial nephritis, interstitial pneumonia, prostatitis and lymphadenopathy (9).

Hepatic IPT was first reported by Pack and Baker (10) in 1953. Although there are several methods for classifying hepatic IPT, such as etiology and tumor location, some authors proposed histological classification. In 1978, Someren (11) classified hepatic IPT into three distinct types: Hyalinized sclerosing, plasma cell granuloma and xanthogranuloma. Later, with the recognition of IgG4-related disease, Zen *et al* (7) classified hepatic IPT (16 cases) into two types, namely fibrohistiocytic (10 cases) and lymphoplasmacytic (6 cases) variants. They reported that lymphoplasmacytic variants could belong to the IgG4-related diseases, while all 16 cases negatively stained with ALK by immunohistochemistry (7). In the present case report, abundant lymphoplasmacytic inflammation illustrating periductal infiltration was observed, but neither xanthogranulomatous inflammation nor multinucleated giant cells was observed, favoring lymphoplasmacytic type. On the other hand, eosinophilic infiltration or IgG4-positive plasma cell infiltration was not remarkable throughout the lesion, while remarkable deposit of hyalinized collagen was observed. These findings are inconsistent with pure lymphoplasmacytic type. More refined histological classification of the hepatic ITP awaits the further accumulation of cases.

The authors could not make a correct diagnosis of this hepatic biliary IPT prior to surgery, and the patient underwent surgery. The imaging analyses strongly suggested intrahepatic CC, and cytodiagnosis of the repeated ENBD bile cytology was malignancy by 5/10 of the cytopathologists/cytotechnologists in our and related institutions. The difficulty in this cytological diagnosis was further verified later by a large number of reviewers (n=42) at the regional slide conference (8). Of note, a core needle biopsy was not performed and the tumor markers for adenocarcinoma were within normal ranges.

Since its first report in 1953(10), the pre-operative diagnosis of hepatic IPT remains very difficult, and surgery is currently performed when malignancy cannot be completely ruled out. A histological confirmation of a biopsy is thus critical for avoiding unnecessary surgery. Some reports have shown the efficacy of the percutaneous core needle biopsy for the diagnosis of hepatic IPT (12); however, Okamoto *et al* (13) reported that hepatic IPT was accurately diagnosed in only 12/23 cases (52.1%) by percutaneous needle biopsy. In addition, ‘seeding’ of the needle tract has been a concern in patients who are surgical candidates (14). Accordingly, cytological techniques have become the initial diagnostic modality when malignant biliary stricture is suspected. Bile aspiration and brush cytology via ERCP drainage tube and repeated ENBD bile cytology are preferably performed. ENBD is simple, repeatable and suitable for comprehensive screening, but diagnosis is often difficult due to low cell recovery rates and the effects of bile acid-induced cytopathies (15). False-positive results for regenerative atypia and false-negative results for low cell recovery rate in well-differentiated CC should also be noted (16). The sensitivity of bile cytology for malignant stricture has been reported to be 30% (28/93) for ERCP aspiration bile cytology, 48% (62/130) for brush cytology and 24% (19/79) for ENBD bile aspiration cytology (17). Low sensitivity of ENBD bile cytology may be overcome by repeated aspiration cytology (1 to 14 times), which increases the diagnostic yield up to 72.3% (34/47), and 32 positive results have been obtained at the maximum of six samplings (18). As far as is known, there is no report of false-positive rates of ENBD bile cytology to date. Moreover, false-negative and false-positive rates in common bile duct brushing cytology during ERCP have been reported to be 13.3% (22/166) and 4.2% (7/166), respectively (19). False-positive results of ERCP brush cytology occur most often in patients with primary sclerosing cholangitis, IgG4-related cholangitis and autoimmune pancreatitis (19,20). Marked periductal inflammation, fibrosis and epithelial degenerative changes can be the cause of atypical cells mimicking malignancy. Taken together, the definitive diagnosis of hepatic biliary IPT may be intrinsically difficult only by ENBD biliary cytology.

Several studies have attempted to identify definite cytologic criteria that can predict malignancy in bile juice. In 1995, Cohen *et al* (21) first reported the cytologic criteria for biliary malignancy. Their analysis revealed that nuclear molding, chromatin clumping and an increased nuclear/cytoplasmic (N/C) ratio, and less importantly, anisonucleosis, nuclear irregularity and nuclear enlargement were frequently associated with malignancy (primary Iowa criteria) (21). In 1998, Renshaw *et al* (22) similarly reported that nuclear molding, chromatin clumping and loss of polarity were associated with malignancy (Boston criteria). In 2002, Henke *et al* (23) successfully applied the Iowa criteria to liquid-based specimens. In 2017, Avadhani *et al* (24) pointed to 11 characteristics significantly associated with malignancy, including 3-dimension clusters, pleomorphism, 2-cell population, hypo/hyper chromasia, a high N/C ratio, cytoplasmic vacuoles, nuclear irregularity, cellular discohesion, hypercellularity, nuclear molding and prominent nucleoli. They concluded that the identification of 3/11 characteristics markedly improves diagnostic performance. In Japan, the criteria proposed by Hirooka *et al* (5) have been used for cytological diagnosis of pooled bile juice (5,6). The rate of accurate diagnosis by these criteria was at most only 61% (5), although it is assumed to be superior to individual criteria of respective cytotechnologists by ~20% (25). These criteria are also used at Ota Memorial Hospital, and the case presented herein was diagnosed as malignant according to the three factors met for A (large clusters) (Table I). It is now deemed that further attention should have been paid to the abnormalities of individual cells in small clusters, such as an increased N/C ratio and nuclear hyperchromasia. However, it was assumed that enlarged nuclei and irregular shaped nuclei (B.1 and 2 in Table I) and enlarged nucleoli may not be useful, as these findings were observed in this IPT case (Fig. 3D).

The final key for differentiation between IPT and CC may be tumor markers. In this case, CA19-9 and CEA levels were both within normal ranges. If these data had been seriously considered and percutaneous core needle biopsy was performed, unnecessary surgery may have been avoided. Of note, CA19-9 levels may be elevated in benign diseases in pancreaticobiliary stricture lesions. In a previous study, out of the 17 cases of hepatic IPT, CA19-9 was elevated beyond its normal range (<37 U/ml), as high as 518.8 U/ml in 4 cases (23.5%) (26). The optimal cut-off value for CA19-9 varies depending on the study. Kuzu *et al* (27) stated that CA19-9 at the cut-off value (72.5 U/ml) was an effective predictive factor in diagnosing tumors in the pancreaticobiliary region. On the other hand, CA19-9 is not produced in patients belonging to the Lewis blood group (α^-^β^-^), which occurs in 5-10% of the population that lacks the enzyme 1,4-fucosyltransferase required for antigen epitope production (28). In such cases, DU-PAN-2 may be an alternative to CA19-9 testing (29). As regards CEA, Juntermanns *et al* (30) reported that only 55 (40%) out of the 136 patients with hilar CC exhibited elevated CEA levels prior to surgery. Thus, normal tumor marker levels do not necessarily exclude malignancy, but it may be a reason to consider needle biopsy.

In conclusion, a case of biliary IPT of the liver was presented herein. Although tumor markers had been within normal ranges and core needle biopsy had not been performed, this case was diagnosed as CC and underwent left hepatic lobectomy based on imaging analyses and ENBD bile cytology. As ENBD bile cytology may play a limited role in differentiation between benign and malignant bile strictures, surgical indication should be carefully determined in patients with normal tumor marker levels.

## Figures and Tables

**Table I:** Diagnostic criteria for bile cytology by the JSCC 2015.

A, Large clusters
1. Irregularly overlapped nuclei
2. Irregularly arranged nuclei
3. Irregular cluster margins
B, Small clusters and isolated cells
1. Enlarged nuclei
2. Irregularly shaped nuclei
3. Abnormal chromatin
C, Other notable findings
1. Necrotic background
2. Varying cell cluster size
D, Point to note
1. Do not make a judgment from limited abnormalities
2. Even if morphological changes occur when bile juice is left as it is, cytological diagnosis may be possible by observing the nuclear structure
3. Benign tumors and normal tissue display cytological features of equal internuclear distance and regularly arranged cytoplasm at cluster margins

Cells or clusters are diagnosed as positive for adenocarcinoma if three factors are met for A or B. This table was reproduced from table 2 in the study by Hirooka *et al* (5) with permission from JSCC. JSCC, Japanese Society of Clinical Cytology.

**Table II:** A multi-reviewer re-evaluation of cytomorphology of the present IPT case and 2 cases of well-differentiated CC.

	Tumor markers		Cytological diagnosis			
Case no. (age, years/sex)	CEA (ng/ml; reference range, 0.0-5.0)	CA19-9 (U/ml; reference range, 0.0-37.0	Benign	Atypia	Suspicious for malignancy	Malignancy
Case 1 (76/F)	2.8	<2.0	0	5	0	5
Case 2 (67/M)	5.4	73.8	0	1	1	8
Case 3 (70/M)	1.9	144.1	1	2	1	6

Case 1 is the present biliary IPT and cases 2 and 3 are well-differentiated CC for comparison. CC, cholangiocarcinoma; CEA, carcinoembryonic antigen; IPT, inflammatory pseudotumor.

## Data Availability

The data generated in the present study are included in the figures and/or tables of this article.
